# Neostigmine-Atropine Combination Triggered Stress Cardiomyopathy in a Healthy Patient: A Case Report and Literature Review

**DOI:** 10.7759/cureus.91738

**Published:** 2025-09-06

**Authors:** Aoumar G Chamma, Wendy E Saliba, Linda Chamma, Samia Metri

**Affiliations:** 1 Cardiology, University of Balamand, Beirut, LBN; 2 Urology, Lebanese University, Beirut, LBN

**Keywords:** atropine, cardiac anesthesia, cardiac arrhythmia and takotsubo cardiomyopathy, induction of ventricular fibrillation, neostigmine, takotsubo cardiomyopathy

## Abstract

Stress-induced cardiomyopathy or Takotsubo syndrome represents a short-term heart condition that develops after experiencing sudden physical or emotional distress. The condition primarily affects postmenopausal women under stress, but drug-induced cases remain both uncommon and poorly identified. A 40-year-old female patient without heart conditions developed stress-induced cardiomyopathy after receiving standard-dose neostigmine-atropine during her elective laparoscopic cholecystectomy. The patient developed ventricular fibrillation, which needed immediate resuscitation just 50 seconds after drug administration. The patient developed Takotsubo cardiomyopathy symptoms with apical ballooning and severe mid-apical hypokinesis according to imaging results, even though coronary angiography showed normal results. The case demonstrates that neostigmine-atropine can cause serious cardiac issues in people with low-risk profiles and suggests using sugammadex as a safer alternative when available.

## Introduction

Surgical procedures require neuromuscular blocking drugs (NMBDs) to establish endotracheal intubation and optimal surgical conditions through deep muscle relaxation [1]. The agents function by blocking nicotinic acetylcholine receptors at the neuromuscular junction to stop depolarization and muscle contraction [2]. The timely and effective reversal of neuromuscular blockade residues remains essential to minimize postoperative pulmonary complications such as respiratory failure or aspiration [3].

Neostigmine, a quaternary ammonium carbamate, functions as the primary acetylcholinesterase inhibitor for reversing nondepolarizing neuromuscular blockade [4]. Neostigmine, by inhibiting acetylcholinesterase activity, leads to elevated synaptic acetylcholine levels, which restore neuromuscular transmission [5]. The increased global acetylcholine levels trigger excessive muscarinic receptor stimulation, which can lead to dangerous bradyarrhythmias, potentially fatal arrhythmias, and asystole [6].

Atropine, on the other hand, blocks the parasympathetic (vagal) action on the heart. Thus, the administration of atropine as an antimuscarinic agent together with neostigmine serves to reduce parasympathetic side effects [7]. The traditional administration of 0.04 mg/kg neostigmine with 0.02 mg/kg atropine is considered safe based on known clinical data [8]. However, the combination of neostigmine and atropine has led to rare incidences of dangerous arrhythmias, which pose safety risks to patients with specific vulnerabilities [9].

Our case describes a previously healthy female patient with no cardiac history, who experienced ventricular fibrillation (VF) after receiving neostigmine-atropine combination treatment, which demonstrates a major yet understated risk.

## Case presentation

A 40-year-old previously healthy woman with a BMI of 27 kg/m² presented for an elective laparoscopic cholecystectomy. Her past medical and social history is unremarkable except for occasional tobacco use (three to five cigarettes weekly). She denied any episodes of syncope, chest pain, or palpitations. Preoperative assessment comprises a 12-lead electrocardiogram (ECG) tracing. As per institutional preoperative protocol, a transthoracic echocardiogram was performed to rule out structural heart disease before an elective laparoscopic cholecystectomy, and it revealed normal findings.

General anesthesia was induced with intravenous propofol (200 mg) and fentanyl (150 µg), followed by rocuronium (50 mg) to facilitate endotracheal intubation. Standard American Society of Anesthesiologists monitoring demonstrated sinus rhythm (78 bpm), blood pressure of 123/68 mmHg, and oxygen saturation of 97% on room air. Anesthesia maintenance was achieved with sevoflurane (1.0 minimum alveolar concentration) in a 50% oxygen/air mixture.

The surgery was uneventful and lasted approximately 50 minutes. Toward the end, train-of-four monitoring showed a recovery ratio of 0.9, suggesting adequate spontaneous neuromuscular recovery. Nevertheless, per institutional protocol, the anesthesia team administered neostigmine 3.2 mg (0.04 mg/kg) together with atropine 1.6 mg (0.02 mg/kg) intravenously as a simultaneous combination. In our center, glycopyrrolate is not routinely available, so atropine is paired with neostigmine for reversal.

Within 50 seconds of reversal agent administration, the patient suddenly developed VF, confirmed on continuous ECG monitoring. Defibrillation was prepared immediately, but chest compressions and oxygenation were initiated as part of standard advanced cardiovascular life support until the defibrillator was charged. A biphasic shock at 200 J was delivered promptly, followed by a second shock and 1 mg epinephrine, with return of spontaneous circulation achieved after four minutes.

**Figure 1:**
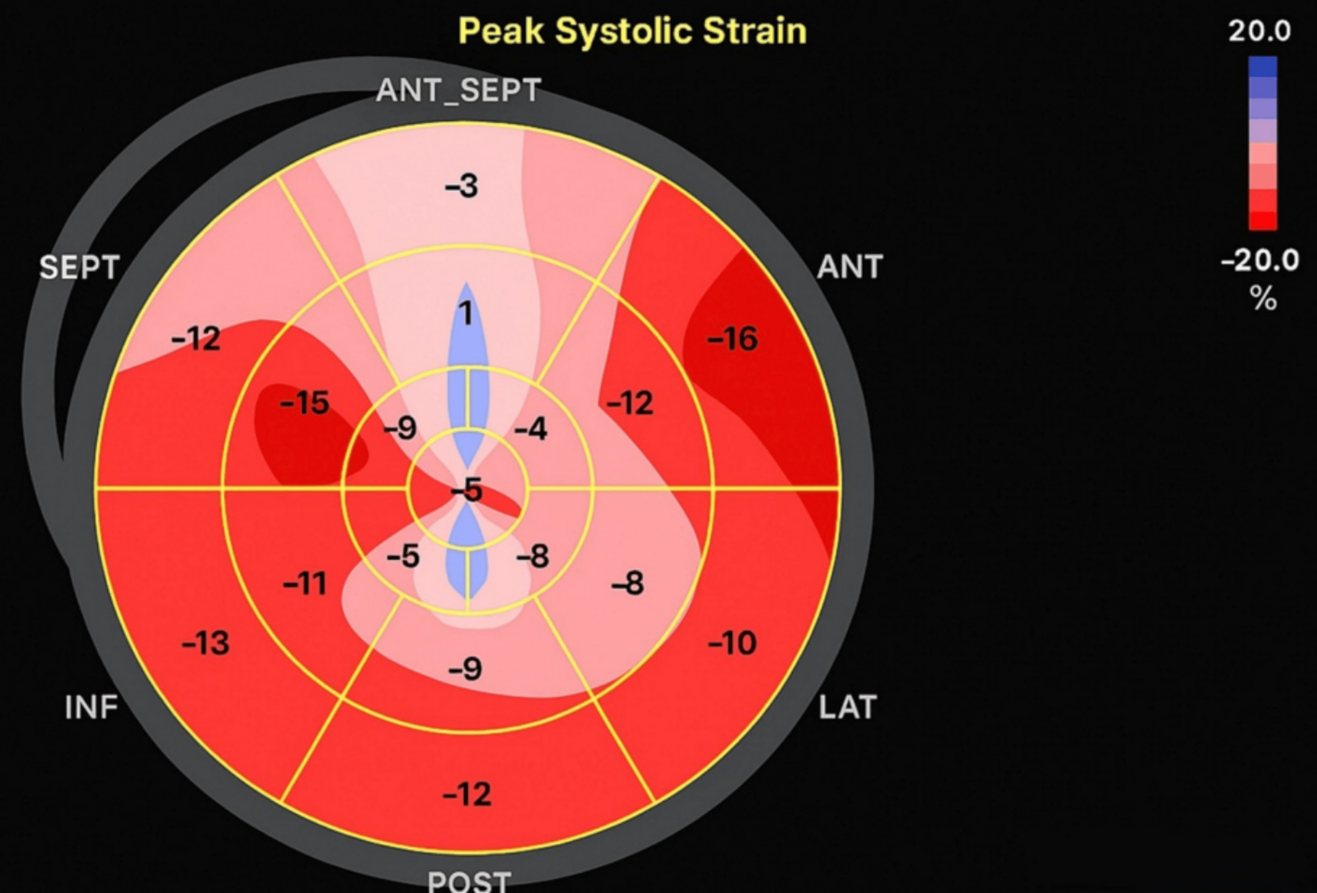
GLS showing apical hypokinesia GLS: global longitudinal strain; ANT: anterior; ANT-SEPT: anteroseptal; INF: inferior; LAT: lateral; POST: posterior

**Figure 2:**
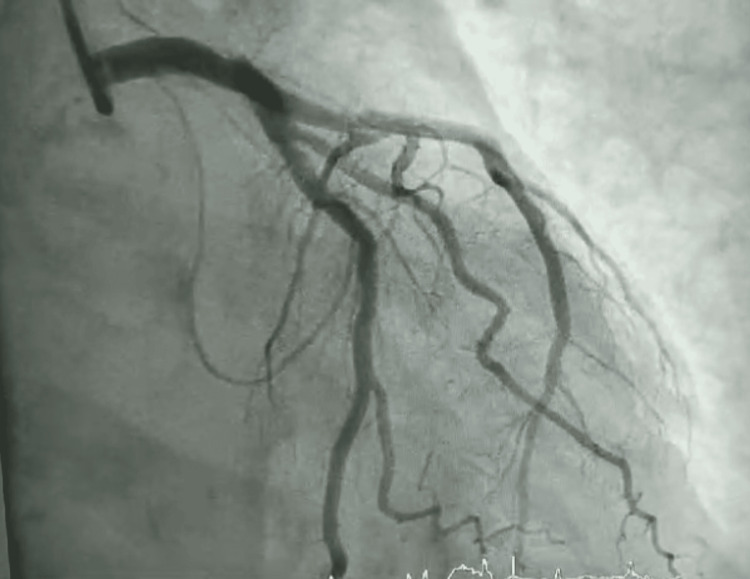
Coronary angiography of the left coronary system with no significant stenosis

**Figure 3:**
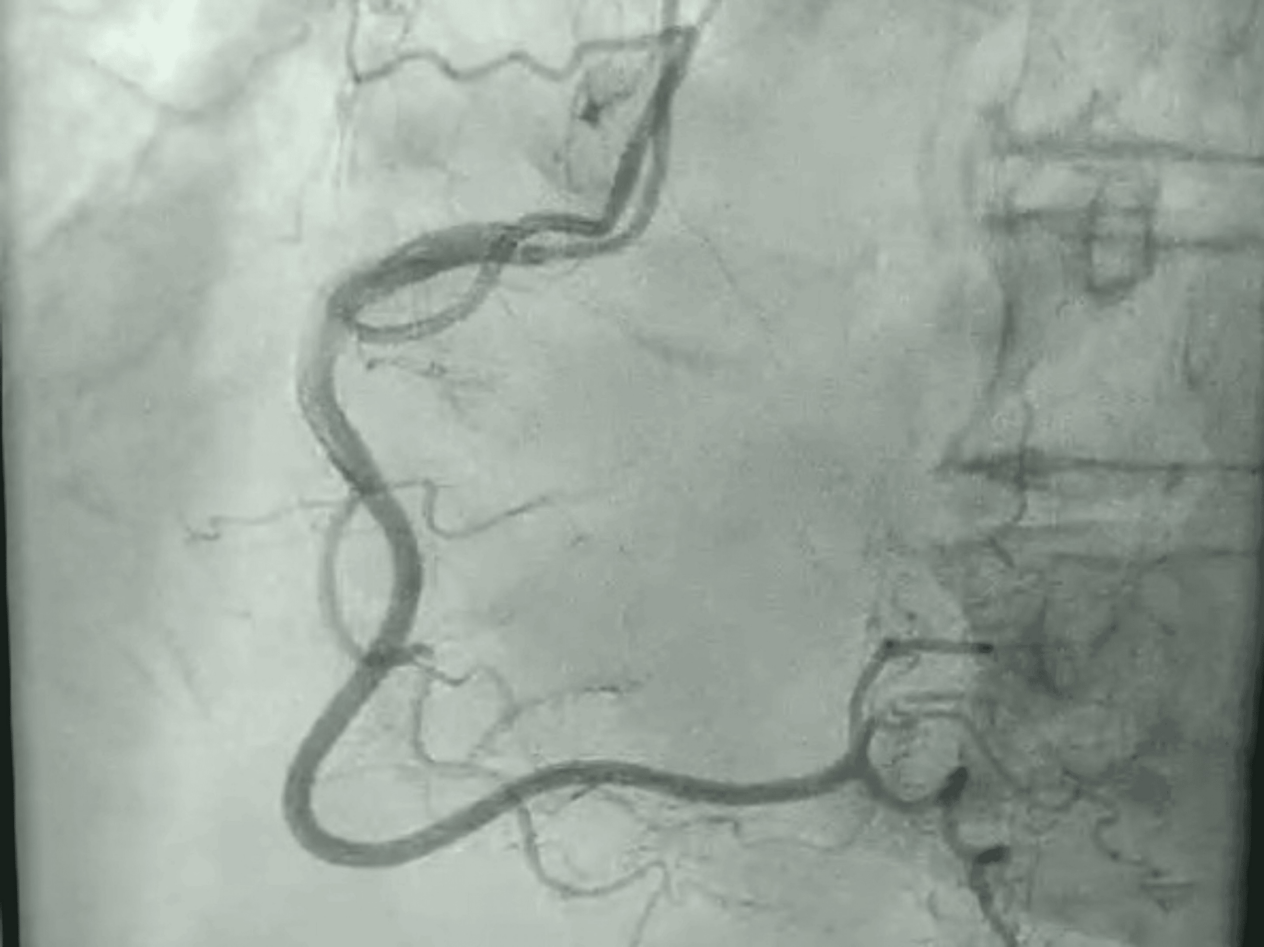
Coronary angiography of the right coronary system with no significant stenosis

**Figure 4:**
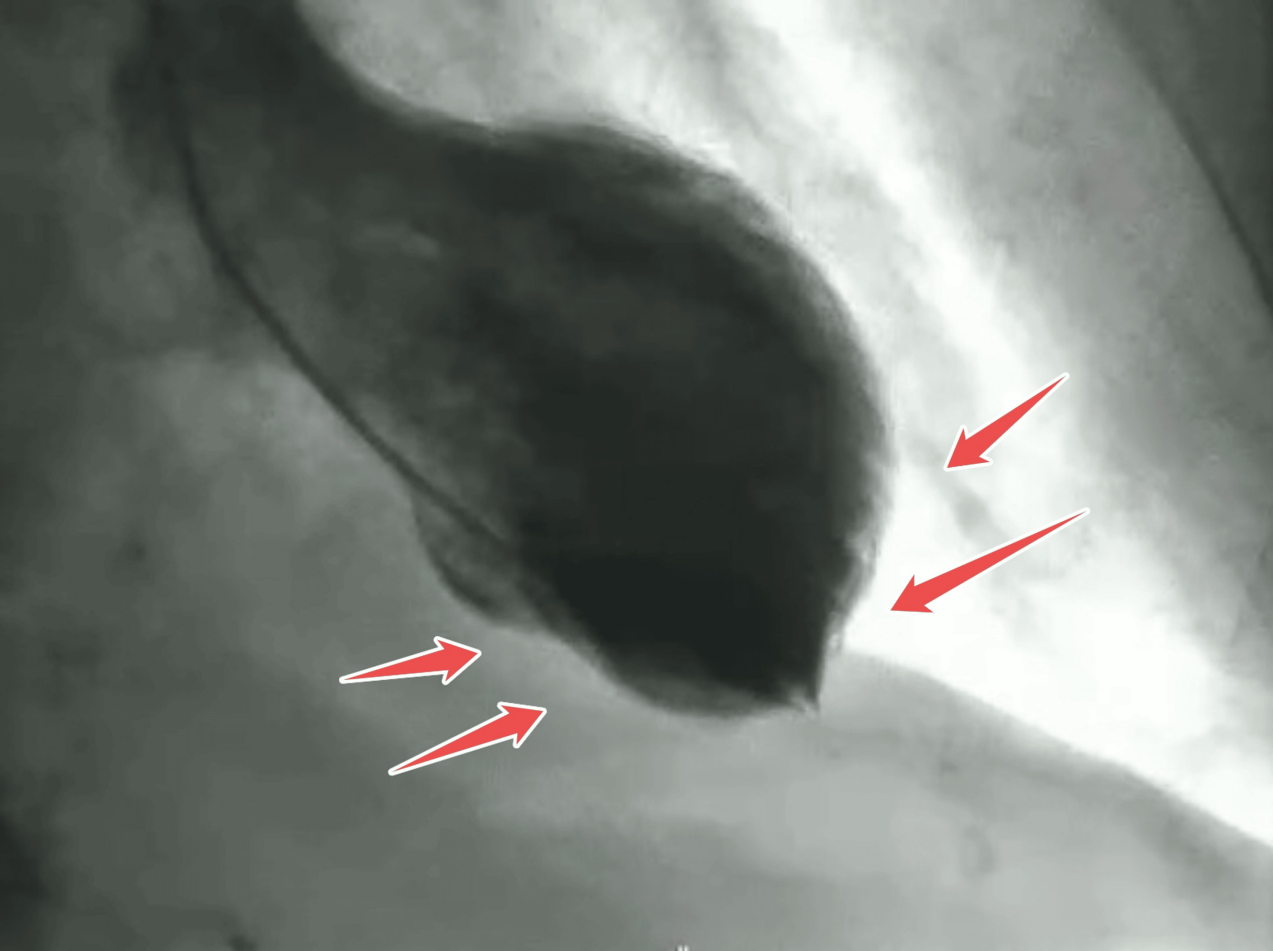
Left ventriculography showing apical ballooning

Postresuscitation echocardiography revealed new-onset severe hypokinesis of the mid to apical left ventricular segments on global longitudinal strain (Figure 1), while coronary angiography performed subsequently showed normal coronary arteries (Figures 2, 3), with a balloon shape on left cardiac ventriculography (Figure 4). Troponin levels were mildly elevated with a value of 0.11 ng/mL (with a reference range of 0.04 ng/mL). The patient was monitored in the intensive care unit and made a complete neurological and cardiac recovery within 72 hours.

## Discussion

This case is significant as it highlights a rare occurrence of VF following neostigmine-atropine administration in a previously healthy patient with no known cardiac history. The majority of previous reports have illustrated bradycardia, junctional rhythms, and rare asystole after neostigmine administration [10].

The main pathophysiological mechanisms at play are likely to be multiple. The inhibition of acetylcholinesterase by neostigmine leads to an increase in acetylcholine, causing profound parasympathetic activation. The muscarinic blocking action of atropine is counteracted by its sympathomimetic effects, which can result in a big change in the balance of the autonomic nervous system. Such autonomic imbalance may produce malignant arrhythmias on a susceptible myocardium especially during the physiological stress of anesthetic emergence [11].

The sudden, rapid onset of VF within 50 seconds after administration makes it less likely that there were other factors at play, such as hypoxia, electrolyte abnormalities, or surgical complications. The transient left ventricular dysfunction seen after the patient's resuscitation was similar to stress-induced cardiomyopathy (Takotsubo), which is a condition that is becoming increasingly recognized after acute catecholamine surges [12].

The use of anticholinesterase-anticholinergic combinations is associated with many risks; therefore, the use of selective relaxant-binding agents such as sugammadex has become more common. Sugammadex, a selective relaxant-binding agent, was precisely developed for the rapid reversal of nondepolarizing neuromuscular blockade induced by rocuronium. It provides many clinical benefits, such as increased patient safety, rapid and predictable reversal of any degree of block, as well as decreased instances of residual block on recovery [13]. Studies have shown that sugammadex ensures a faster reversal of rocuronium‐induced neuromuscular block compared to neostigmine, no matter the depth of the block. Sugammadex 2 mg/kg is 10.22 minutes and almost 6.6 times more rapid in reversing moderate neuromuscular blockade than 0.05 mg/kg neostigmine [13]. Sugammadex 4 mg/kg is 45.78 minutes and almost 16.8 times more rapid in reversing deep neuromuscular blockade than 0.07 mg/kg neostigmine [13]. Additionally, sugammadex appears to have a better safety profile than neostigmine [13]. Studies have shown that patients receiving sugammadex demonstrate fewer adverse events compared to those given neostigmine [13].

Sugammadex incorporates aminosteroidal NMBDs (e.g., rocuronium) without altering acetylcholine metabolism; thus, there is no risk of autonomic imbalance [14]. Sugammadex has been shown to reduce the incidence of postoperative arrhythmias compared to neostigmine in recent meta-analyses [15].

This case presents an unusual instance of VF that occurred after a standard neostigmine-atropine dose administration to a healthy patient. The fast development of VF during the first 50 seconds after drug administration indicates that the neostigmine-atropine combination triggered this condition. The patient recovered from resuscitation but developed stress-induced cardiomyopathy without coronary artery disease, which demonstrated the severe nature of this adverse drug reaction. These findings call for a reevaluation of the routine use of neostigmine, particularly in low-risk individuals with near-complete spontaneous neuromuscular recovery. Anesthesiologists should evaluate the need for neostigmine administration in these situations while considering the implementation of sugammadex as a treatment option when available.

## Conclusions

This case report demonstrates that standard doses of neostigmine-atropine can trigger dangerous ventricular arrhythmias in people with normal heart function. The administration of neuromuscular blockade reversal necessitates critical cardiac monitoring. Future guidelines need to establish risk stratification methods prior to selecting reversal agents.
